# Nanocomposites based on lanthanide-doped upconversion nanoparticles: diverse designs and applications

**DOI:** 10.1038/s41377-022-00871-z

**Published:** 2022-07-13

**Authors:** Kaimin Du, Jing Feng, Xuan Gao, Hongjie Zhang

**Affiliations:** 1 State Key Laboratory of Rare Earth Resource Utilization, Changchun Institute of Applied Chemistry, Chinese Academy of Sciences, Changchun, 130022 Jilin, China; 2 State Key Laboratory of Molecular Reaction Dynamics, Dalian Institute of Chemical Physics, Chinese Academy of Science, 116023 Dalian, China; 3 University of Science and Technology of China, Hefei, Anhui 230026 China; 4 Department of Chemistry, Tsinghua University, 100084 Beijing, China

**Keywords:** Nanoparticles, Nonlinear optics

## Abstract

Lanthanide-doped upconversion nanoparticles (UCNPs) have aroused extraordinary interest due to the unique physical and chemical properties. Combining UCNPs with other functional materials to construct nanocomposites and achieve synergistic effect abound recently, and the resulting nanocomposites have shown great potentials in various fields based on the specific design and components. This review presents a summary of diverse designs and synthesis strategies of UCNPs-based nanocomposites, including self-assembly, in-situ growth and epitaxial growth, as well as the emerging applications in bioimaging, cancer treatments, anti-counterfeiting, and photocatalytic fields. We then discuss the challenges, opportunities, and development tendency for developing UCNPs-based nanocomposites.

## Abstract (web summary)

This review presents a summary of diverse designs and applications of nanocomposites based on lanthanide-doped upconversion nanoparticles.

## Graphical abstract

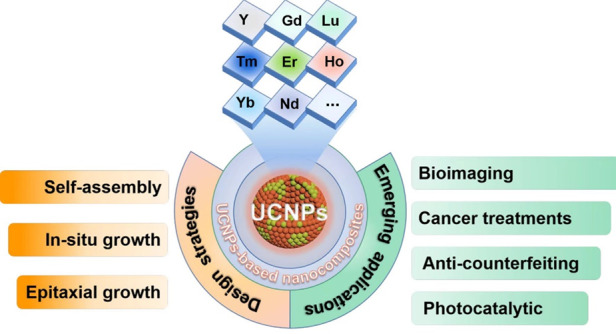

## Introduction

**Fig. 1:**
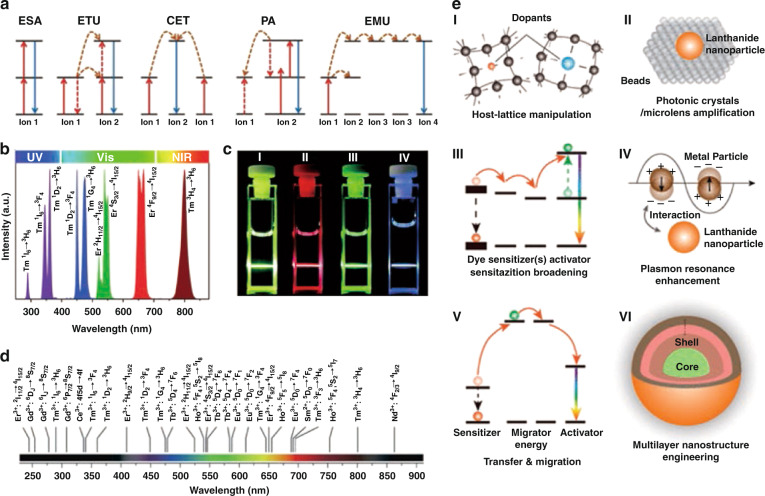
Upconversion processes and optical modulation. **a** Schematic diagrams of five upconversion processes^4^. **b** Representative UCL emissions of UCNPs doped with Yb–Er and Yb–Tm ranging from UV to NIR region under irradiation at 980 nm. **c** Photographs of UCL of UCNPs in colloidal solution. (I) Total UCL of NaYF_4_:Yb,Er sample. (II, III) The UCL of NaYF_4_:Yb,Er sample through red and green filters, respectively. (IV) Total UCL of NaYF_4_:Yb,Tm sample^3^. **d** By selecting the proper type of doping Ln^3+^ within UCNPs, a broad range of emission wavelengths from UV to NIR spectral region that could be modulated^5^. **e** Several photoluminescence enhancement strategies in Ln^3+^-doped UCNPs. (I) host lattice modulation, (II) photonic crystal and microlens magnification, (III) molecular sensitization, (IV) plasmon resonance enhancement, (V) energy transfer/transfer manipulation, (VI) core–shell engineering^7^. **a** Reprinted with permission from ref. ^4^ Copyright 2020, Elsevier. **b**, **c** Reprinted with permission from ref. ^3^ Copyright 2015, The Royal Society of Chemistry. **d** Reprinted with permission from ref. ^5^ Copyright 2019, Elsevier. **e** Reprinted with permission from ref. ^7^ Copyright 2020, American Chemical Society.

Lanthanide-doped upconversion nanoparticles (UCNPs) which can absorb two or more low-energy photons and radiate a high-energy photon are favored by researchers in various fields^1,2^. The 4*f* electrons of rare-earth ions constitute the rich metastable energy levels, which makes the upconversion process of rare-earth-doped nanocrystals diversified. In general, the upconversion luminescence (UCL) processes associated with rare-earth ions mainly involve five types of energy transfer (ET) pathways: excited state absorption (ESA), energy transfer upconversion (ETU), cooperative energy transfer (CET), photon avalanche (PA), and energy migration upconversion (EMU), as shown in Fig. 1a^3,4^. In UCNPs, lanthanide ions (Ln^3+^) with ladder-like electronic energy levels are often co-doped as activators for UCL process. Before in-depth understanding of EMU process, efficient activators were limited to Er^3+^, Tm^3+^, and Ho^3+^ (especially Er^3+^ and Tm^3+^). For UCNPs co-doped with Yb^3+^–Er^3+^, the energy transitions of ^2^H_11/2_ → ^4^I_15/2_, ^4^S_3/2_ → ^4^I_15/2_, and ^4^F_9/2_ → ^4^I_15/2_ occurred under 980 nm excitation, green (525 nm and 542 nm) and red (655 nm) UCL were observed. For Tm^3+^ ions, under 980 nm excitation, ultraviolet (UV) emissions (290, 345, and 362 nm), visible (Vis) emissions (450, 475, 644, and 694 nm) and near infrared (NIR) emission (800 nm) could be observed. The UCL spectral of Er^3+^ and Tm^3+^ in UCNPs are shown in Fig. 1b, and the photograph of UCL is shown in Fig. 1c^3^. As the EMU process is proposed, the UCL of rare-earth ions (Ce^3+^, Gd^3+^, Tb^3+^, Dy^3+^, Eu^3+^, Sm^3+^, and Sm^2+^ ions) without suitable intermediate energy levels has been realized through the construction of core–shell structures (Fig. 1d)^5,6^. The modulation of emission wavelengths in UV-to-NIR spectral region could be easily achieved by selecting appropriate Ln^3+^ within UCNPs. As known, Ln^3+^ have inherent limitations such as narrow absorption cross-sections and inefficient nonlinear multiphoton processes. Many strategies, including host lattice modulation, photonic crystal and microlens magnification, molecular sensitization, plasmon resonance enhancement, energy transfer/transfer manipulation, core–shell engineering, etc., are currently used to overcome these difficulties (Fig. 1e)^7^. Reassuringly, compared with conventional materials with downshifting luminescence (DSL), such as luminescent complexes, organic dyes, and quantum dots (QDs), upconversion materials have excellent physical, chemical and biological characteristics, such as narrow-band emission, long fluorescence lifetime, large anti-Stokes shifts, superior light and chemical stability, low biological toxicity, and so on^8–12^. Due to the above advantages, UCNPs have a wide range of applications in three-dimensional display^13,14^, solar spectrum conversion^15^, anti-counterfeiting technology^16^, optical sensing^17^, and biomedicine^18–20^.

On account of the restricted features, single nanomaterial is often unable to meet the needs of practical applications. During the last decade, the design and fabrication of multifunctional nanocomposites have aroused immense research interests. Particularly, scientists have made much effort to develop UCNPs-based nanocomposites, which are composed of UCNPs and other functional materials. In terms of design and synthesis, self-assembly and in-situ growth are usually used to obtain UCNPs-based nanocomposites with core/satellite structures. In addition, UCNPs-based nanocomposites with core–shell structure could be prepared by epitaxial growth. In terms of multifunctional properties, UCNPs-based nanocomposites often retain the unique photoluminescence properties of UCNPs, and could be endowed with variable properties of other various functional materials. In terms of applications, combine UCNPs with bioimaging contrast agents, chemotherapy drugs, photothermal agents, photodynamic agents, and chemodynamic agents could be used for diagnosis and treatment of malignant tumor; UCNPs integrated with other fluorescent materials are potential candidates for multi-modal anti-counterfeiting; The integration of UCNPs and semiconductor photocatalysts have great potentials in NIR light-induced photocatalysis.

In this review, we firstly summarize the synthesis methods of UCNPs-based nanocomposites for various design purposes, i.e., self-assembly (electrostatic adsorption, specific recognition reaction, and covalent bonding), in-situ growth, and epitaxial growth. Then, we systematically introduce the applications of such nanocomposites, i.e., bioimaging, cancer treatments (chemotherapy, photothermal therapy, photodynamic therapy, synergistic cancer therapeutics), anti-counterfeiting, and photocatalysis. Finally, we discuss the challenges, future directions, and suggestions for UCNPs-based nanocomposites.

## Strategies toward design and synthesis of UCNPs-based nanocomposites

To integrate UCNPs and other functional materials into one nanosystem, many strategies have been developed. Herein, we review the recent studies on the construction strategies and synthesis approaches of UCNPs-based nanocomposites, mainly classified into three categories: self-assembly, in-situ growth, and epitaxial growth.

### Self-assembly

Self-assembly methods play an important role in the construction of multifunctional nanocomposites, mainly preparing various monomer components in advance and then combining such different components to form one nanosystem^21^. Recently, the common self-assembly methods for preparing UCNPs-based nanocomposites primarily contain electrostatic adsorption, specific recognition reaction, and covalent bonding.

#### Electrostatic adsorption

**Fig. 2:**
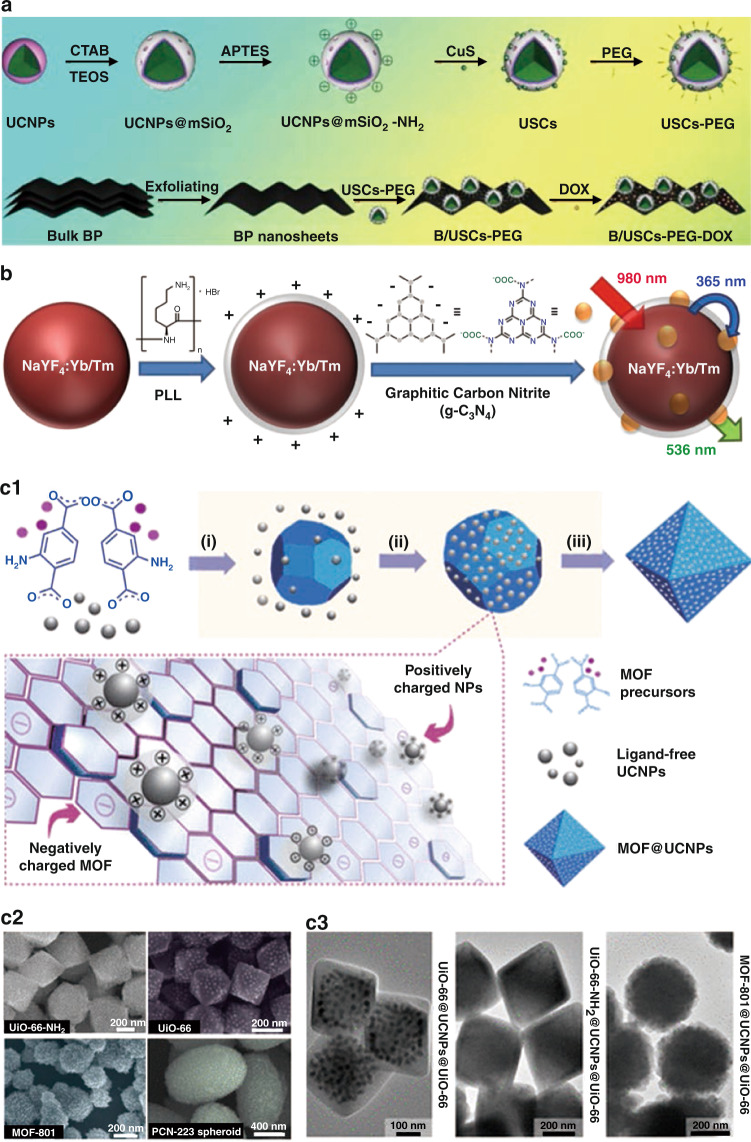
Electrostatic adsorption technique. **a** Schematic diagram of the synthetic process of B/USCs-PEG-DOX^24^. **b** Schematic illustration of the preparation of NaYF_4_:Yb/Tm-PLL@g-C_3_N_4_^25^. **c1** Schematic of the synthetic process of UCNPs and MOF nanocomposites. **c2** SEM images of UiO-66-NH_2_@NaYF_4_:Yb/Er, UiO-66@NaYF_4_:Yb/Er, MOF-801@NaYF_4_:Yb/Er, PCN-223@NaYF_4_:Yb/Er nanocomposites. **c3** TEM images of MOF@UCNPs@MOF sandwiched nanocomposites^26^. **a** Reprinted with permission from ref. ^24^ Copyright 2020, Elsevier. **b** Reprinted with permission from ref. ^25^ Copyright 2016, American Chemical Society. **c1–c3** Reprinted with permission from ref. ^26^ Copyright 2018, American Chemical Society

Electrostatic adsorption is a typical strategy for the synthesis of nanocomposites. Specifically, nano-monomers with different charges were synthesized separately, and then combined through electrostatic interaction to form nanocomposites^22–27^. Shi et al. ^22^ reported a core/satellite nanocomposite by decorating negatively charged CuS nanoparticles onto positively charged UCNPs@SiO_2_–NH_2_ nanoparticles. Yang et al.^24^ developed an intelligent nanoplatform by conjugating mesoporous silica (mSiO_2_)-coated UCNPs (UCNPs@mSiO_2_) with CuS nanoparticles and black phosphorus (BP) nanosheets. In Fig. 2a, positively charged UCNPs@mSiO_2_–NH_2_ were obtained by (3-aminopropyl)triethoxysilane (APTES) modification. Then, CuS was conjugated further through electrostatic adsorption and PEG was introduced to improve the water solubility. Finally, BP nanosheets with negative charges were coupled with UCNPs@mSiO_2_–CuS–PEG (USCs–PEG). This study takes full advantage of electrostatic adsorption to integrate several components with different charge modifications into one nanoplatform. Liu et al. ^25^ reported a novel NaYF_4_:Yb/Tm-PLL@g-C_3_N_4_ nanoplatform. In Fig. 2b, NaYF_4_:Yb/Tm core was decorated with positive ligand poly(l-lysine) (PLL). Graphitic carbon nitride (g-C_3_N_4_) with negatively charged COO^−^ groups could combine with NaYF_4_:Yb/Tm-PLL through electrostatic interaction. Recently, new nanomaterials have been developed by combining metal-organic frameworks (MOFs) with UCNPs^26,27^. Huang et al. ^26^ used electrostatic interactions to spread UCNPs onto MOFs surfaces (Fig. 2c1). Various MOFs such as UiO-66-NH_2_, UiO-66, MOF-801, and PCN-223 are paved with UCNPs through this method (Fig. 2c2). Furthermore, MOF@UCNPs@MOF with sandwich structure could be obtained through the epitaxial growth of MOF (Fig. 2c3). This work provides a simple route to construct the nanocomposites combined MOFs and UCNPs with unique structures.

#### Specific recognition reaction

**Fig. 3:**
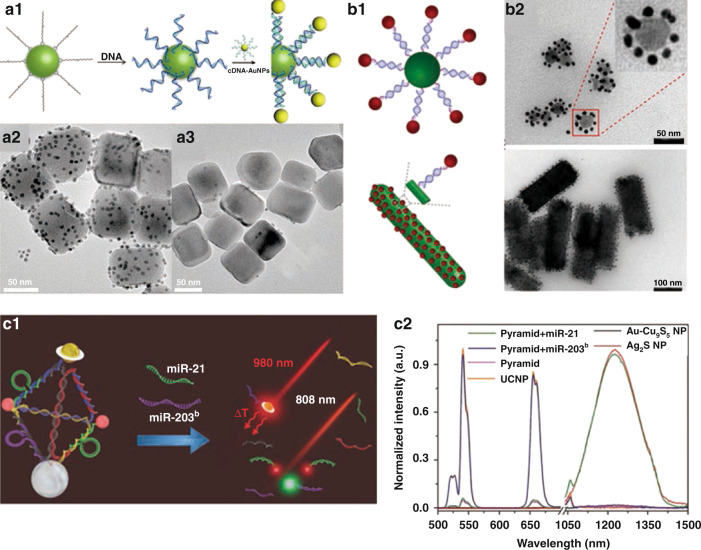
Specific recognition reaction. **a1** Schematic illustration of DNA-directed assembly of UCNPs and AuNPs. TEM images of the assembly of T30-UCNPs with AuNPs containing complementary DNA (**a2**) and non-complementary DNA (**a3**)^32^. **b1** Schematic illustrations of the DNA-mediated assemblies of UCNPs and AuNPs (5 nm). **b2** TEM images of DNA-mediated assemblies UCNPs (NaYF_4_:Yb/Er nanospheres and NaYF_4_:Gd/Yb/Er nanorods) with AuNPs^33^. **c1** Nanopyramids for dual miRs detection. **c2** Luminescence spectra of Ag_2_S NPs, Au-Cu_9_S_5_ NPs, UCNPs, pyramids, pyramids with miR-21, and pyramids with miR-203^b^ excited by 808 nm laser^34^. **a1–a3** Reprinted with permission from ref. ^32^ Copyright 2013, American Chemical Society. **b1**, **b2** Reprinted with permission from ref. ^33^ Copyright 2020, John Wiley and Sons. **c1**, **c2** Reprinted with permission from ref. ^34^ Copyright 2017, John Wiley and Sons

Specific recognition reaction mainly uses certain specific biological molecules (DNA and RNA aptamers^28,29^, avidin and biotin^30^, antigens and antibodies^31^, etc.) to combine multiple components together. DNA is an ideal programmable self-assembling agent due to its precise length and well-known Watson–Crick base pairing. Lu et al. ^32^ fabricated DNA-modified UCNPs directly by a simple method, and then integrated with AuNPs modified with complementary DNA strand. In Fig. 3a1, T30 oligonucleotides modified UCNPs (T30-UCNPs) could assemble with complementary DNA strand modified AuNPs (A27-AuNPs). The satellite structure could be constructed by surrounding T30-UCNPs with several A27-AuNPs (Fig. 3a2). In contrast, when non-complementary DNA-modified AuNPs were incubated with T30-UCNPs, the self-assembly could not be achieved owing to lack of specific recognition between the two parts (Fig. 3a3). This work proposes a novel combinatorial approach and further extend the applications in biomimetic nano-assembly and biomedicine. Huang et al. ^33^ proved the molecular recognition and programmable assembly capabilities of the as-prepared monodispersed DNA-UCNPs by mixing them with DNA-AuNPs together. DNA strands on UCNPs and AuNPs will hybridize to form the duplex, forming the satellite-like structure with UCNPs in the center and AuNPs outside (Fig. 3b1). AuNPs can be uniformly coated on NaYF_4_:Yb/Er nanospheres and NaYF_4_:Gd/Yb/Er nanorods through DNA-guided assembly (Fig. 3b2). The obtained nanocomposites could realize the adjustment of the distance between nanoparticles, which provides new thought for the subsequent synthesis of other nanocomposites. Kuang et al. ^34^ assembled nanopyramids with Au–Cu_9_S_5_, UCNPs, and Ag_2_S by using the complementary DNA hybridization between the recognition sequences and DNA skeleton (Fig. 3c1). When miR-21 and miR-203^b^ appeared, the recognition sequences connected to Ag_2_S and UCNPs in the pyramid were completely complementary and separated from DNA framework based on competitive hybridization. Two luminescent signals could be restored simultaneously under 808 nm excitation, which arise from UCNPs at 541 nm and Ag_2_S at 1227 nm in the Vis and second window of NIR (NIR-II) region, respectively (Fig. 3c2). This proposed strategy opens extensive opportunities for self-assembled nanostructures in the biological field.

#### Covalent bonding

In some UCNPs-based nanocomposites, UCNPs are coupled with other components through amidation reaction, in which the two parts containing functional groups could share electron pairs to form stable covalent bonds. In the amidation reaction, 1-ethyl-3-(3’-dimethylaminopropyl)carbodiimide (EDC) and N-hydroxysuccinimide (NHS) are usually served as cross-linking agents to activate the carboxyl group (EDC/NHS method)^35–38^. Our group^35^ constructed ZnO-gated UCNPs@mSiO_2_ nanoplatform. EDC was acted as the cross-linking agent to facilitate the formation of amide bonds between carboxylic acid-functionalized UCNPs@mSiO_2_ and amine-capped ZnO nanodots, enabling ZnO nanodots to be firmly anchored to UCNPs surface. Shan et al. ^36^ covalently combined amino-functionalized UCNPs with carboxylated nanodiamond by using EDC and NHS as cross-linking agents to prepare multifunctional nanoplatform. Compared with the electrostatic adsorption, the nanocomposites obtained by covalent bonding have high structural stability and biocompatibility.

### In-situ growth

**Fig. 4:**
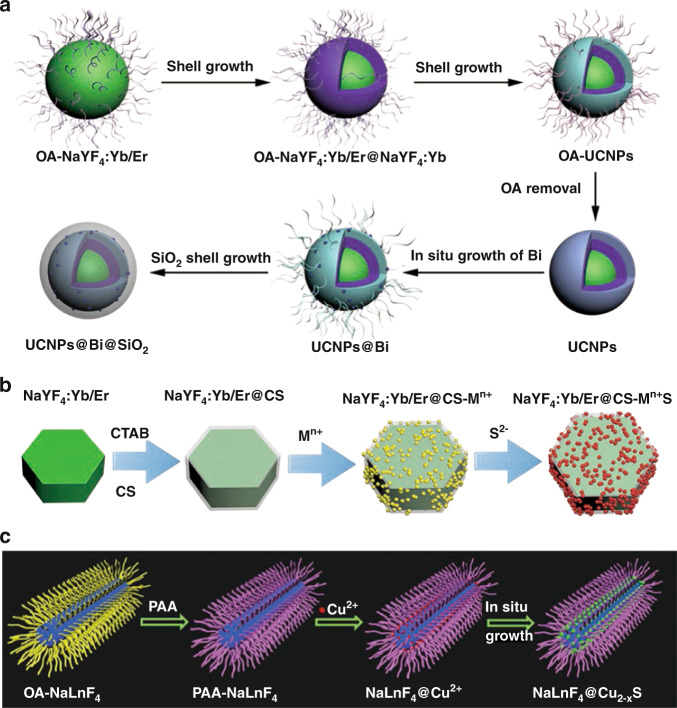
In-situ growth strategy. **a** Schematic diagram of synthetic process of UCNPs@Bi@SiO_2_ nanocomposites^41^. **b** Schematic illustration of preparation of NaYF_4_:Yb/Er@CS@M^n+^S nanocomposites^43^. **c** Schematic illustration of designing NaLnF_4_@Cu_2-x_S theranostic nanoplatform^47^. **a** Reprinted with permission from ref. ^41^ Copyright 2019, American Chemical Society. **b** Reprinted with permission from ref. ^43^ Copyright 2020, The Royal Society of Chemistry. **c** Reprinted with permission from ref. ^47^ Copyright 2019, John Wiley and Sons

In-situ growth strategy is mainly summarized as a method of first synthesizing one of the components, and then uniform growing the other component to its surface^39–49^. Up to now, most nanocomposites based on UCNPs and single “element” nanoparticles (such as Au and Bi) have been synthesized through in-situ growth strategy^39–41^. You et al. ^41^ used in-situ growth method to synthesize UCNPs@Bi and finally obtain UCNPs@Bi@SiO_2_ nanocomposites through surface modification of SiO_2_. Oleic acid (OA) coated UCNPs were first obtained and ligand-free UCNPs were prepared through the acid-induced ligand removal process. Then, Bi nanoparticles could be well decorated on UCNPs through in-situ growth. Finally, the outermost layer of UCNPs@Bi nanoparticles is coated with dense SiO_2_ shell to improve the stability (Fig. 4a). Nowadays, conjugating transition-metal chalcogenide (M^n+^S/Se) and UCNPs has aroused immense research interests^42–47^. Chitosan (CS) with glucosamine and hydroxyl groups could chelate metal ions (Ag^+^, Cu^2+^, Cd^2+^, etc.) and improve the biocompatibility of UCNPs. Our group^43^ reported a general in-situ growth method that can make (M^n+^S, M = Ag, Cu, Cd) nanodots to uniformly conjugate on CS-coated UCNPs (Fig. 4b). First, OA-modified NaYF_4_:Yb/Er UCNPs were prepared and transferred into hydrophilic phase with the assistance of cetyltrimethylammonium bromide (CTAB). Then, CS was introduced to further immobilize M^n+^. Finally, M^n+^S QDs could be well decorated on UCNPs@CS through in-situ growth after adding the sulfur source. Such facile in-situ growth strategy could also be used to combine Ag_2_Se nanodots with CS-coated UCNPs, only using selenium source instead of sulfur source^44^. Besides, Hao et al. ^47^ synthesized NaLnF_4_@Cu_2-x_S nanocomposites using in-situ growth strategy (Fig. 4c). OA-coated NaLnF_4_ was firstly synthesized by hydrothermal method. The oil-phase core could be transferred to the water phase and negatively charged by PAA modification. Then, Cu^2+^ could be absorbed by PAA-NaLnF_4_. Finally, Cu_2−*x*_S grow uniformly in-situ on NaLnF_4_ by adding sulfur source. For in-situ growth strategy, polymers or complexes are firstly modified on UCNPs, which will then act as nucleation and growth centers to induce further growth of other nanodots. This method simplifies the multi-step additional loading steps, and could control the growth behavior of nanodots on UCNPs by adjusting the feeding ratio.

### Epitaxial growth

Growing homogeneous shell on UCNPs through epitaxial growth is generally considered to be an effective approach to reduce the original nanocrystal surface defect density, thereby improving the UCL efficiency. Various core–shell UCNPs have been constructed, such as inert core–shell structures (NaYF_4_:Yb/Ln@NaYF_4_ (Ln = Er, Tm)^50–52^, NaGdF_4_:Yb/Tm@NaGdF_4_^53,54^, LaF_3_:Yb/Tm@LaF_3_^55^) or active core–shell structures (NaYF_4_:Yb/Tm@NaYF_4_:Yb/Er^56^, NaGdF_4_:Yb/Er@NaGdF_4_:Yb^57^, NaGdF_4_:Yb/Tm@NaGdF_4_:Eu^58^, and BaGdF_5_:Yb/Er@BaGdF_5_:Yb^59^). The heterogeneous core–shell structure refers to the different host lattices between the core and shell, which has been proven to be an effective strategy to form hybrid nanostructures^60–69^. Yan et al. ^61^ found that the UCL efficiency of NaYF_4_:Ln^3+^@CaF_2_ nanoparticles with heterogeneous core–shell structure could be increased by more than 300 times compared with shell-free NaYF_4_:Ln^3+^ nanoparticles. This indicates that heterogeneous core–shell structure can effectively prevent the influence of nanoparticle surface defects and environmental quenching effects. Han et al. ^62^ found that UCL intensity of NaYF_4_:(20–100%)Yb/Tm@CaF_2_ was enhanced by two orders of magnitude compared with the nanoparticles without CaF_2_ shell. Yang et al. ^63^ developed α-NaYbF_4_:Tm@CaF_2_:Nd@ZnO nanoplatform via hetero-epitaxial growth manner. CaF_2_ shell grow epitaxially on α-NaYbF_4_:Tm core, which can not only effectively control the particle size, but also greatly promote UCL intensity. Cubic ZnO layer firmly assembled on α-NaYbF_4_:Tm@CaF_2_:Nd via the epitaxy manner to form core–shell–shell nanostructure. Gao et al. ^64^ epitaxially grown ZnS on KMnF_3_:Yb/Er based on the high affinity of Mn^2+^ for chalcogen ions. The formation of KMnF_3_:Yb,Er@ZnS enhances UCL by suppressing the surface-quenching effects. The core–shell particles also exhibit intense DSL of Mn^2+^ under UV excitation, attributing to Mn^2+^ doping into the ZnS lattice through the core–shell interface.

## Emerging applications of UCNPs-based nanocomposites

Scientific interest in UCNPs-based nanocomposites has rapidly increased and some newly emerging sectors have seen the applications of them including bioimaging, cancer treatments, anti-counterfeiting, photocatalysis, etc.

### Bioimaging

**Fig. 5:**
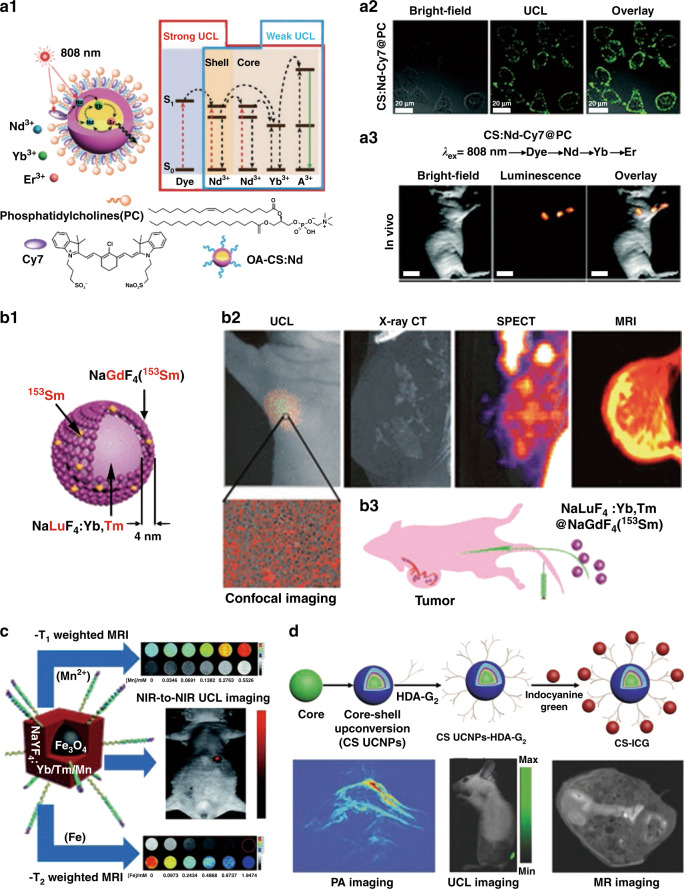
Bioimaging. **a1** Schematic diagram and energy level diagram of PC modified dye-sensitized UCNPs-based nanocomposite for photon upconversion upon 808 nm irradiation. **a2** UCL images of HeLa cells incubated with CS:Nd-Cy7@PC. **a3** UCL lymphatic imaging 30 min after injection of CS:Nd-Cy7@PC under 808 nm excitation^70^. **b1** Composition of NaLuF_4_:Yb,Tm@NaGdF_4_(^153^Sm). **b2** Four-modal imaging of the tumor-bearing nude mouse at 1 h post intravenous injection of NaLuF_4_:Yb,Tm@NaGdF_4_(^153^Sm). **b3** Schematic diagram of tumor angiogenesis imaging using NaLuF_4_:Yb,Tm@NaGdF_4_ (^153^Sm) as the probe^75^. **c** *T*_1_/*T*_2_-weighted MR and NIR-to-NIR UCL imaging of Fe_3_O_4_@NaYF_4_:Yb/Tm/Mn nanoplatform^76^. **d** Schematic of the synthesis of ICG modified UCNPs (CS-ICG) and the PAI, UCL imaging, and MRI of CS-ICG nanocomposites in vivo^83^. **a1–a3** Reprinted with permission from ref. ^70^ Copyright 2016, The Royal Society of Chemistry. **b1–b3** Reprinted with permission from ref. ^75^ Copyright 2013, American Chemical Society. **c** Reprinted with permission from ref. ^76^ Copyright 2017, The Royal Society of Chemistry. **d** Reprinted with permission from ref. ^83^ Copyright 2016, John Wiley and Sons

UCNPs with good chemical and optical stability, low toxicity and good biocompatibility could normally be radiated by NIR light, which have higher tissue penetration depth than short-wavelength UV or Vis light and avoid interference from organism background fluorescence^70–74^. Li’s group^70^ designed phosphatidylcholine (PC) modified dye-sensitized UCNPs-based nanocomposite for biological UCL imaging. A sulfonic functionalized cyanine dye derivative (Cy7) attached on NaYF_4_:30%Yb,1%Nd,0.5%Er@NaYF_4_:20%Nd (CS:Nd) was used as the antenna dye, which could broadly harvest NIR energy and enhance the UCL (Fig. 5a1). The intense UCL signal could be detected in HeLa cells incubated with CS:Nd-Cy7@PC nanocomposite (Fig. 5a2). Moreover, CS:Nd-Cy7@PC was successfully applied in lymphatic imaging (Fig. 5a3), suggesting the feasibility of dye-sensitized upconversion nanocomposite for bioimaging. However, optical imaging still has the limits of resolution and three-dimensional reconstruction. It is necessary to combine other imaging technologies widely used in the medical field, such as magnetic resonance (MR) imaging (MRI), X-ray computed tomography (CT), single-photon emission computed tomography (SPECT), and photoacoustic imaging (PAI), to improve the accuracy and sensitivity of imaging. UCNPs-based nanocomposites offer great opportunities for multi-modal imaging. Especially, UCNPs themselves could achieve various imaging such as UCL imaging, CT imaging, MRI, etc. by modulating lanthanide elements and structure of UCNPs. Li et al. ^75^ developed NaLuF_4_:Yb,Tm@NaGdF_4_:^153^Sm as an optimized multi-modal imaging probe, in which Lu was used for CT imaging, Tm was used for UCL imaging, Gd was applied for MRI and radioisotope ^153^Sm for improving SPECT imaging (Fig. 5b1–b3). It is worth noting that the desired sensitivity and resolution in vivo could be greatly improved by combining MRI and UCL imaging. UCNP-based nanocomposites for UCL/MR dual-modal imaging have been well studied^76–80^. Yang et al. ^76^ synthesized Fe_3_O_4_@Mn^2+^-doped NaYF_4_:Yb/Tm nanoplatform, which can not only offer contrast signal in *T*_1_/*T*_2_-weighted MRI due to the co-existence of Fe_3_O_4_ and Mn^2+^, but also exhibit pronounced NIR-to-NIR UCL (Yb^3+^–Tm^3+^) in vivo fluorescence imaging, (Fig. 5c). As known, MRI is more proper for soft tissue examination, while CT shows great advantages in bone, lung, and chest imaging, as well as cancer detection. Therefore, the combination of MRI, CT, and UCL imaging can undoubtedly provide more comprehensive information of tissues^81,82^. Li’s group^81^ developed Fe_3_O_4_@NaLuF_4_:Yb,Er/Tm nanocomposites as multi-modal (MR, CT and UCL) imaging probe, which could provide the detailed imaging information of tumor-bearing mice. NaGdF_4_:Yb,Er–Ag hybrid nanocomposites were designed for UCL/CT/MR multi-modal bioimaging^82^. PAI with characteristics of non-invasiveness, rapidness, and accurate quantification has attracted immense attention specifically in diagnosis of tumor pathophysiological status. Indocyanine Green (ICG) exhibits strong PAI signal at low concentration owing to the strong absorbance in the range of 740–800 nm^83,84^. Nie et al. ^83^ synthesized high-efficiency UCNPs with 800 nm excitation, and then ICG was loaded onto UCNPs to enhance the total absorption and UCL, achieving PAI and UCL imaging, as well as MRI (Fig. 5d).

### Cancer treatments

How to achieve accurate treatment of cancer has always been a difficult problem and research hotspot in the medical field. After careful modification, UCNPs could be used as promising carriers of multiple functional probes (such as chemotherapeutic agents, NIR photothermal agents, photosensitizer, Fenton nanocatalysts, etc.) for cancer treatments.

#### Chemotherapy

**Fig. 6:**
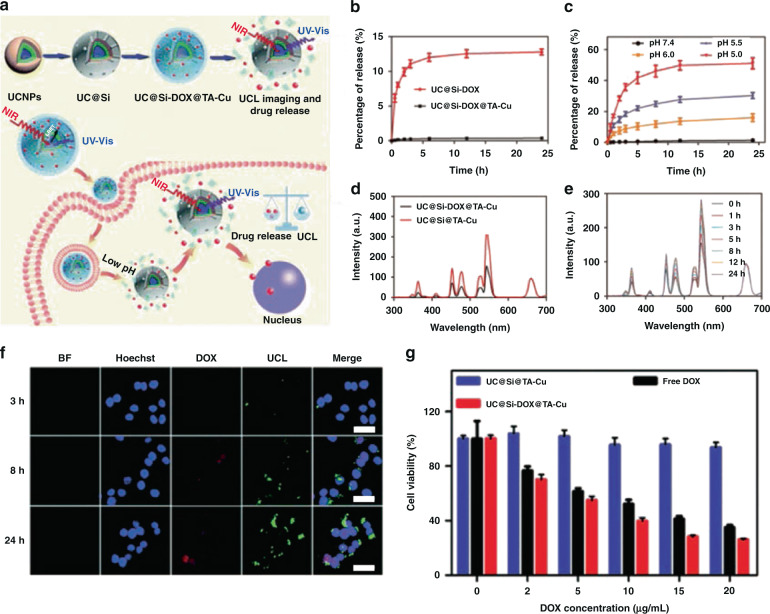
Chemotherapy. **a** Schematic illustration of the fabrication of UC@Si-DOX@TA–Cu and pH-responsive drug release monitored by UCL imaging in real time. **b** Drug release profiles for UC@Si-DOX@TA–Cu and UC@Si-DOX in phosphate buffer saline (PBS) at pH 7.4. **c** Drug release profiles for UC@Si-DOX@TA–Cu in PBS at pH 7.4, 6.0, 5.5 and 5.0. **d** The UCL spectra of UC@Si@TA–Cu and UC@Si-DOX@TA–Cu. **e** Time-dependent UCL spectra of UC@Si-DOX@TA–Cu after immersing in PBS solution (pH = 5.5). **f** Confocal fluorescence images of HeLa cells after incubation with UC@Si-DOX@TA–Cu for 3, 8 and 24 h. Scale bar: 40 μm. **g** Viability of HeLa cells incubated with UC@Si@TA–Cu, DOX and UC@Si-DOX@TA–Cu at varying concentrations^94^. Reprinted with permission from ref. ^94^ Copyright 2019, The Royal Society of Chemistry

Chemotherapy is the way of destroying cancer cells with one or more anti-cancer drugs, which have achieved great success in improving the prognosis of patients. However, its severe toxic side effects are life-threatening, which present new challenges to people to alleviate the risk^85,86^. Various drug vectors such as polymer micelles^87^, vesicles^88^, and inorganic nanoparticles^89^ have been extensively studied in drug loading to reduce adverse reactions. Chemotherapeutic applications of UCNP-based nanocomposites have been studied extensively^90–98^. Our group^35^ designed ZnO-gated UCNPs@mSiO_2_@DOX (DOX = doxorubicin) nanoplatform for specific pH-triggered on-demand drug release. The “gatekeeper” ZnO exists stably in normal tissue environment to prevent premature drug leakage and decomposes in the acidic tumor microenvironment to realize pH-triggered on-demand release of DOX. Li et al. ^94^ designed UC@Si-DOX@TA–Cu (TA = tannic acid) nanoplatform to realize real-time UCL monitoring of pH-responsive drug release in live cells (Fig. 6a). UC@Si-DOX exhibits rapid release behavior at pH 7.4 while UC@Si-DOX@TA–Cu shows negligible DOX leakage, which indicates that TA–Cu protective shell can effectively encapsulate DOX within the mesopores (Fig. 6b). For UC@Si-DOX@TA–Cu, DOX release increases with decreasing pH, attributing to the efficient decomposition of TA–Cu complexes under acidic condition (Fig. 6c). Moreover, the luminescence resonance energy transfer from UCNPs to DOX occurs, resulting in the emission quenching of UCNPs (Fig. 6d). Upon pH triggered DOX release, the luminescence resonance energy transfer is gradually eliminated, resulting in an increase in UCL to monitor DOX release in real-time (Fig. 6e). The real-time monitoring of intracellular DOX release from UC@Si-DOX@TA–Cu exhibits that time-dependent enhancement of DOX fluorescence could be detected with the extension of incubation time. The significant recovery of UCL can be clearly detected under 980 nm excitation (Fig. 6f). The cytotoxicity assay proves that UC@Si-DOX@TA–Cu shows good cancer therapeutic effect (Fig. 6g).

#### Photothermal therapy

Photothermal therapy (PTT) is an efficient noninvasive therapy approach, based on the photothermal conversion effect of photothermal agents to increase the temperature of tumor site for killing the tumor cells under the irradiation of external light. Compare with UV and Vis light, NIR light possesses much higher tissue penetration ability^99^. To date, various organic or inorganic NIR-absorbed photothermal agents have been developed for PTT^100–109^.

NIR light-activated nanocomposites combining UCNPs with photothermal agents show great prospects in PTT of tumor^45,110–115^. Our group^44^ reported multifunctional UCNPs@CS@Ag_2_Se nanocomposites for multi-modal imaging-guided PTT of tumor. Benefiting from the excellent absorption coefficient of Ag_2_Se, the nanocomposites exhibit high photothermal conversion efficiency, and excellent photothermal killing effect. You et al. ^45^ designed UCNP–Bi_2_Se_3_ nanohybrid for UCL/CT imaging-guided PTT. Bi_2_Se_3_ nanodots connected on UCNPs possess strong NIR absorption and efficient photothermal performance as well as distinct cancer cell ablation under single-wavelength NIR laser irradiation. Additionally, Shi et al. ^110^ reported novel UCNP@Al(OH)_3_/Au nanohybrids for synergistic-targeted PTT and UCL imaging under NIR light irradiation.

#### Photodynamic therapy

Photodynamic therapy (PDT) is a noninvasive and site-specific cancer treatment based on photochemistry. Typical PDT system involves three major elements: photosensitizer, suitable excitation of light, and oxygen molecules at the site of the disease tissue. Upon light excitation at specified wavelength, photosensitizer could be selectively activated to generate reactive oxygen species (ROS), which can induce cancer cell death, nevertheless, its application is limited by the penetration depth of the excitation light^116^.

**Fig. 7:**
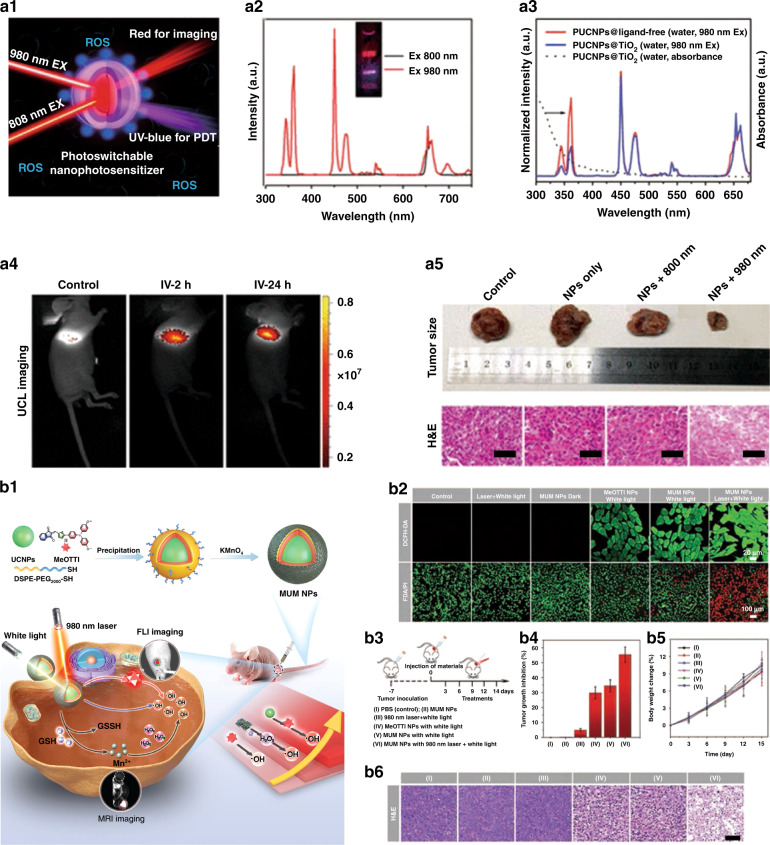
Photodynamic therapy. **a1** Schematic of PUCNPs@TiO_2_ nanocomposites for imaging guided PDT. **a2** Upconversion emission spectra of PUCNPs excited by 800 or 980 nm laser (5 W cm^−2^). Inset: luminescence photograph of PUCNPs in cyclohexane under irradiation by two laser beams (0.8 W cm^−2^). **a3** UCL spectra of PUCNPs@ligand-free (red line) and PUCNPs@TiO_2_ (blue line), the absorbance spectra of PUCNPs@TiO_2_ (dotted line). **a4** UCL imaging in LLC tumor-bearing mouse after intravenous injection of PUCNPs@TiO_2_ for 2 and 24 h (800 nm irradiation). **a5** The digital photographs of excised tumors after various treatments and H&E-stained slices of tumor tissues collected from different groups. The scale bars stand for 50 μm^118^. **b1** Schematic illustration of fabrication of MUM NPs and dual-modal imaging guided triple-jump PDT. **b2** DCFH-DA assay for intracellular ROS level (upper row) and FDA/PI assay for live/dead cell staining (lower row) of 4T1 cells after different treatments. **b3** Schematic diagram of the operation process of antitumor therapy. **b4** Tumor inhibition ratios of different groups after various treatments on day 14. **b5** Body weight profiles of mice under different treatments. **b6** H&E staining analysis of tumor tissues collected from different groups^127^. **a1–a5** Reprinted with permission from ref. ^118^ Copyright 2018, American Chemical Society. **b1–b6** Reprinted with permission from ref. ^127^ Copyright 2021, John Wiley and Sons

As UCNPs could convert NIR light to UV/Vis light, the tissue penetration depth in their PDT application could be improved. Based on this, UCNPs-based nanocomposites become the ideal candidates for PDT of deep tissue cancer^117–124^. Kong et al. ^118^ designed NIR light switchable PUCNPs@TiO_2_ nanocomposites to realize imaging guided accurate PDT of tumor (Fig. 7a1). PUCNPs (NaErF_4_@NaYF_4_@NaYbF_4_:0.5%Tm@NaYF_4_) possess the superior photoswitching feature. The intense UV-blue emission of Tm^3+^ could be observed under 980 nm excitation while it could be completely turned off when irradiated by 800 nm light. Additionally, red emission could be detected under 800 nm laser irradiation, attributing to the multi-wavelength excitation (800, 980, and 1530 nm) property of NaErF_4_ (Fig. 7a2). Using this switching characteristic, TiO_2_ photosensitizer could absorb UV light selectively resulting from PUCNPs. The ET efficiency between PUCNPs and TiO_2_ is calculated as 63% based on the UV/red emission ratio (Fig. 7a3). In vivo results show that the upconversion photoswitching nanocomposites are appropriate for UCL imaging in real-time and PDT of cancer under spatio-temporal control (Fig. 7a4, a5). This work promotes the application of UCNPs optical switching nanomaterials in biological field. Tang et al. ^119^ developed switchable DNA/UCNPs nanocomposite with chlorin e6 (Ce6) functionalization, which could produce singlet oxygen (^1^O_2_) and perform effective PDT for cancer under 980 nm excitation, providing new insights for precise targeting and highly efficient cancer therapy. At present, photosensitizers with aggregation-induced emission (AIE) features do not need to rely on O_2_ and have better therapeutic effects on the hypoxic regions of tumors^125^. However, AIE-active photosensitizers could often be irradiated by UV light with limited tissue penetration and the preparation of AIE-active photosensitizers with long-wavelength optical windows tends to be very complicated^126^. To optimize the diagnosis and treatment performance of AIE active photosensitizers, Tang et al. ^127^ constructed tumor microenvironment-responsive multifunctional nanoplatform (MUM NPs), which is composed of AIE-active photosensitizer (MeOTTI), UCNPs, and MnO_2_. The fluorescence resonance energy transfer (FRET) between UCNPs and MeOTTI could extend the wavelength of excitation light from UV–Vis to NIR region, which greatly enhances the tissue penetration depth and hydroxyl radicals (•OH) production efficiency. In the tumor microenvironment, MnO_2_ shells could decompose and effectively reduce the expression of intracellular glutathione (GSH), thereby increasing the level of intracellular •OH. Mn^2+^ generated by the reaction can catalyze the generation of •OH from intracellular H_2_O_2_, and finally realize the “triple jump” of ROS level and effective PDT guided by UCL/MR imaging (Fig. 7b1). Both the intracellular ROS generation assay and the corresponding live–dead staining assay demonstrated the extraordinary PDT performance of MUM NPs (Fig. 7b2). MUM NPs can strongly inhibit tumor proliferation and destroy tumor tissue, achieving highly effective antitumor therapy (Fig. 7b3–b6). This work inspires researchers to further explore therapeutic nanoplatforms with diversified AIE-active photosensitizers for prospective clinical translation.

#### Synergistic cancer therapeutics

Except chemotherapy, PTT and PDT mentioned above, UCNPs-based nanocomposites could also be applied for radiotherapy (RT), chemodynamic therapy (CDT), gas therapy as well as immunotherapy. In order to better control tumor progression and prevent tumor metastasis and recurrence, the combination of multiple treatments has become an inevitable trend in cancer treatment.

**Fig. 8:**
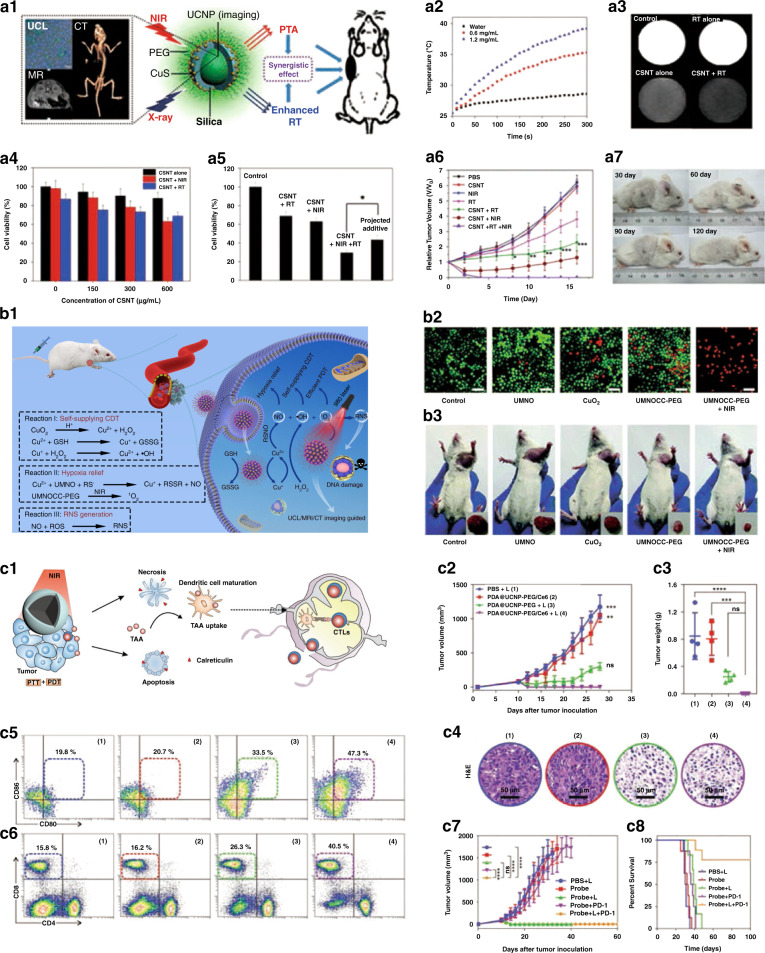
Synergistic cancer therapeutics. **a1** Schematic illustration of CSNT for imaging-guided RT/PTA synergistic therapy. **a2** Temperature variation of CSNT solutions irradiated by a 980 nm NIR laser (1.5 W cm^−2^, 5 min). **a3** The impact of CSNT on the X-ray radiation dose. **a4** Viability of HeLa cells incubated with CSNTs at different concentrations with or without 980 nm laser irradiation and RT. **a5** Viability of HeLa cells that have taken up CSNTs treated with RT, PTA, and RT/PTA. **a6** Relative tumor growth curves of different groups. **a7** Digital photographs of mice from group 7 after 30, 60, 90, and 120 days of treatment^22^. **b1** Schematic illustration of UMNOCC-PEG for imaging-guided tumor therapy. **b2** CLSM images of HeLa cells co-stained with calcein AM (live cells, green) and PI (dead cells, red) after different treatments (0.5 W cm^−2^, 500 μg mL^−1^). **b3** Photographs of the representative mice and excised tumors^137^. **c1** Schematic illustration of synergistic phototherapy to enhance antitumor immunity. Tumor growth curve (**c2**), tumor weight (**c3**), and representative H&E staining (**c4**) of 4T1 tumor-bearing mice after different treatments. Detection of DC maturity (CD80^+^CD86^+^ gated on CD11c^+^) in tumor-draining lymph nodes (**c5**) and CTLs (CD4^−^CD8^+^ gated on CD3^+^) in the spleen (**c6**) by flow cytometry. Mean tumor growth kinetics (**c7**) and corresponding survival rates (**c8**) of mice after different treatments^139^. **a1–a7** Reprinted with permission from ref. ^22^ Copyright 2013, American Chemical Society. **b1–b7** Reprinted with permission from ref. ^137^ Copyright 2020, The Royal Society of Chemistry. **c1–c8** Reprinted with permission from ref. ^139^ Copyright 2019, John Wiley and Sons

RT is one of the most commonly used therapies using high energy electromagnetic radiation to inhibit tumor growth, which has better results in patients who cannot undergo surgical treatment or have difficult resection^128,129^. As known, the combination of RT and PTT will counteract the disadvantages of PTT alone for deep tumors. To improve therapeutic effect of cancer by integrating photothermal ablation (PTA) with RT, Shi et al. ^22^ designed UCNPs@SiO_2_@CuS (CSNT) nanocomposites for UCL/CT/MR imaging guided RT/PTA synergistic therapy (Fig. 8a1). Such nanocomposites possess distinct photothermal conversion performance under 980 nm excitation through the adherence of CuS satellites (Fig. 8a2). CSNT can be used as a radiation sensitizer to produce dose enhancement effect due to the existence of high *Z* elements (Yb, Gd, and Er) (Fig. 8a3). The applicability of CSNT as efficient photothermal conversion agent and radiosensitizer was well demonstrated (Fig. 8a4, a5). Through the synergistic therapeutic effect of RT/PTA, tumor tissue could be completely eradicated without late recurrence (Fig. 8a6, a7), laying a foundation for future early diagnosis and multi-modal imaging-guided synergistic tumor therapy.

CDT, as a burgeoning type of technology for tumor treatment, has aroused great research interest. CDT takes the acidic microenvironment in the tumor as the reaction condition, over expressed H_2_O_2_ as the reaction raw material and transition metal nanomaterials as the catalyst to trigger Fenton or Fenton-like reaction in cancer cells, catalyze H_2_O_2_ to produce highly cytotoxic •OH and induce irreversible mitochondrial damage, DNA strand breakage, and protein and membrane oxidation^130–132^. Lin et al. ^133^ designed UCNPs–Pt(IV)–ZnFe_2_O_4_ nanoplatform for collaborative PDT/CDT/chemotherapy of cancer. NaGdF_4_:Yb/Tm@NaGdF_4_:Yb UCNPs triggered by NIR light could serve as UV–Vis light source to induce PDT effect and Fenton reaction of ZnFe_2_O_4_. Pt(IV) prodrugs could be reduced to highly toxic Pt(II) through GSH in tumor cells. This nanoplatform provide a comprehensive way for synergetic anticancer therapy. Besides, our group^134^ designed Cu_2−*x*_S decorated NaYF_4_:Yb/Er@NaYF_4_:Yb UCNPs to achieve synergistic enhanced CDT/PTT of cancer.

Gas therapy is promising for the treatment of many diseases due to its inherent biosafety and insignificant side effects. So far, gaseous molecules including NO, H_2_, H_2_S, SO_2_, and CO have shown significant anticancer effect^135–137^. Yang et al. ^137^ reported a versatile Cu^2+^-initiated NO nanotheranostic system (UMNOCC–PEG) for UCL/CT/MR imaging guided CDT/PDT/gas combination therapy. When UMNOCC–PEG nanocomposite is endocytosed by tumor cells, pH-sensitive CuO_2_ nanodots are decomposed, allowing the release of Cu^2+^ ions and H_2_O_2_. This not only triggers the Fenton-like reaction of Cu^2+^ and H_2_O_2_, but also realizes efficient CDT by solving the problem of limited endogenous H_2_O_2_ content. It can alleviate the antioxidant capacity and hypoxia of tumor through NO production and GSH consumption, to further boost the therapeutic effect of CDT and PDT (Fig. 8b1). In vitro and in vivo experimental results demonstrate that UMNOCC–PEG has excellent synergistic anticancer ability (Fig. 8b2, b3).

Immunotherapy is a treatment mode to improve the intrinsic ability against tumor by activating the body’s own immune system, which can not only effectively inhibit tumor recurrence and metastasis, but also specifically kill tumor cells that have relapsed and metastasized^138–140^. Liu and co-authors synthesized polydopamine (PDA) coated with NaGdF_4_:Yb/Er shell, and then loaded the photosensitizer Ce6 on its surface (PDA@UCNP–PEG/Ce6)^139^. The nanocomposites could elicit robust systemic and humoral antitumor immune responses by synergistic phototherapy (Fig. 8c1). The synergistic treatment group (group 4) could effectively eradicate tumors (Fig. 8c2–c4), confirming that synergistic phototherapy performed better than PDT or PTT alone in tumor ablation. The cell maturation efficacy and cytotoxic T lymphocytes (CTLs) in the spleen in the synergistic treatment group were much higher than those in the control group (Fig. 8c5, c6), proving that synergistic phototherapy could induce systemic antitumor immune response. Importantly, the combination of PDA@UCNP–PEG/Ce6 and PD-1 blocking antibody can effectively inhibit tumor recurrence and metastasis (Fig. 8c7) and prolong the survival period of tumor-bearing mice (Fig. 8c8). This study establishes an innovative paradigm for increasing immunogenic cell death by synergistic phototherapeutic nanoplatforms.

### Anti-counterfeiting applications

Counterfeit and shoddy currencies, drugs and valuables are increasingly damaging to the market economy, bringing immeasurable economic loss to consumers and copyright holders. Therefore, a variety of anti-counterfeiting materials such as digital watermark, diffraction grating, photonic structure, stimulus response materials and luminescent materials have been developed and widely used in the current anti-counterfeiting technology. However, the security level of traditional anti-counterfeiting materials is relatively low and easy to be copied^141–143^.

**Fig. 9:**
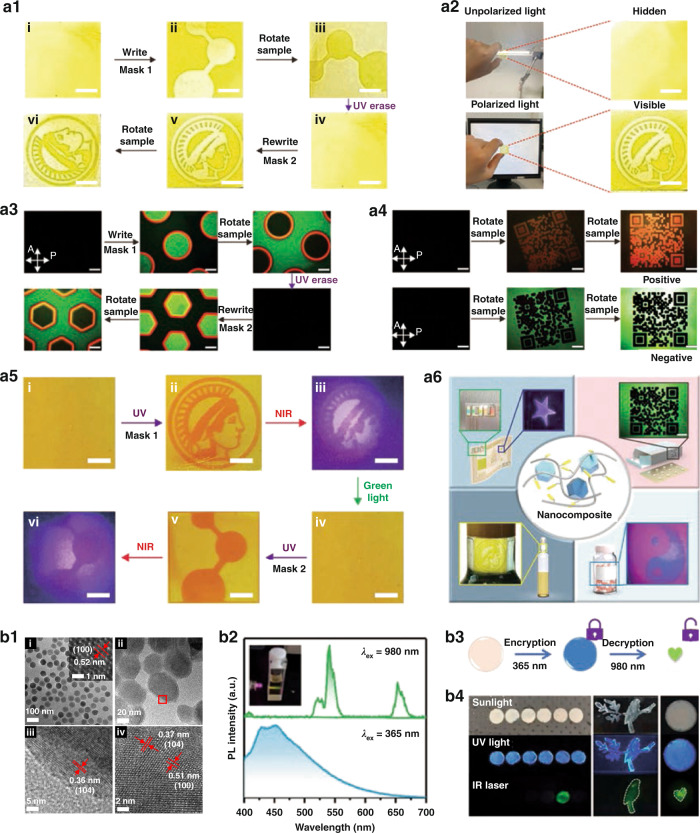
Anti-counterfeiting applications. **a1** Photographs of erasable and rewritable macropolarization-related patterns of nanocomposite film. Scale bars stand for 0.5 mm. **a2** Photographs of macroscopic polarization-dependent patterns under unpolarized and polarized light. Scale bars stand for 0.5 mm. **a3** Polarization microscope photographs of microscopic polarization correlation patterns. Scale bars: 200 μm. **a4** QR code pattern on different backgrounds under polarized light microscope. Scale bars: 200 μm. **a5** Photographs of photochromic UCL patterns of PAzo/UCNPs under NIR light. Scale bars stand for 5 mm. **a6** PAzo/UCNPs nanocomposite for different anti-counterfeiting applications^150^. **b1** (i) TEM image of NaYF_4_:Yb^3+^,Er^3+^ UCNPs. (ii) TEM image of UCNP@CsMnCl_3_ nanocomposites. (iii) The magnified TEM images enclosed by the red frame in (ii). (iv) High-resolution image of UCNP@CsMnCl_3_ recorded by spherical aberration electron microscopy. **b2** Photoluminescence spectra of UCNP@CsMnCl_3_ excited by 980 nm laser and 365 nm UV light. Inset: luminescence photograph of UCNP@CsMnCl_3_ under irradiation by 980 nm laser. **b3** Dual-modal light-emitting anticounterfeiting principle diagram. **b4** Images of security patterns made of pure CsMnCl_3_ and UCNP@CsMnCl_3_ under diverse excitation modes (sunlight, UV light, NIR laser)^158^. **a1–a6** Reprinted with permission from ref. ^150^ Copyright 2021, John Wiley and Sons. **b1–b4** Reprinted with permission from ref. ^158^ Copyright 2021, John Wiley and Sons

Lanthanide-doped UCNPs are especially suitable for anti-counterfeiting because of their rich intermediate state energy levels and distinguishable spectral characteristics^144–149^. Multicolor dual-modal excitation can be realized simultaneously by controlling the species and distribution of Ln^3+^ ions in the core and shells or developing new nanocomposites in combination with other luminescent materials^150–152^. Recently, Wu et al. ^150^ introduced UCNPs in photoresponsive azobenzene-containing polymer (azopolymer) to form PAzo/UCNPs nanocomposites, which have the characteristics of various anti-counterfeiting manners and read-out methods, as well as easy processing. First, different color patterns were prepared by using the photoisomerization properties of PAzo/UCNPs. Based on the differences of mechanical features between *trans*- and *cis*-azopolymers, the periodic arranged photonic crystal structures could be obtained by embossing. Due to Bragg diffraction, a pattern with structural color is finally presented. According to photoinduced orientation properties of azopolymers, macroscopic and microscopic polarization related patterns were further prepared (Fig. 9a1–a4). Because *cis*-azopolymers can absorb upconversion blue light, the photochromic pattern can be recognized under irradiation of NIR light according to the synergistic effect of azopolymers and UCNPs (Fig. 9a5). According to practical needs of anti-counterfeiting, the nanocomposites could be coated with various patterns on flexible substrates and applied in banknotes, medicine boxes, wine bottles, and medicine bottles (Fig. 9a6). The research work is important for designing the high-end anti-counterfeiting materials.

Recently, a series of nanocomposites have been developed by coupling UCNPs with perovskite quantum dots, which play the important roles in multi-modal anti-counterfeiting^153–158^. Our group^153^ designed UCNPs–CsPbX_3_ (UCX_3_) nanocomposites, emitting multicolor UCL/DSL under 980 nm laser or 365 nm lamp excitation. In addition, UCX_3_/polystyrene with multicolor fluorescence and dual-modal luminescence features could be used as a quick drying fluorescent ink for writing and printing, which greatly increase the difficulty of fraud and provide insight for the practical application in anti-counterfeiting. Lin et al. ^158^ synthesized UCNP@CsMnCl_3_ nanocomposites, which play an important role in high-quality optical anti-counterfeiting. NaYF_4_:Yb^3+^,Er^3+^ was used as core, followed by heteroepitaxial growth of CsMnCl_3_ to obtain the core–shell structure (Fig. 9b1). UCNP@CsMnCl_3_ exhibits the characteristic UCL of Er^3+^ (980 nm excitation, Fig. 9b2, top). Broad blue DSL of CsMnCl_3_ is realized upon excitation with 365 nm light (Fig. 9b2, bottom). To verify the anti-counterfeiting capability, CsMnCl_3_ and UCNP@CsMnCl_3_ are used initially to prepare dot-based patterns. Fig. 9b3 shows the relevant encryption and decryption process, and Fig. 9b4 shows the three anti-counterfeiting modes based on this design. The decryption cannot be completed under sunlight and UV light irradiation. Under excitation at 980 nm, only UCNP@CsMnCl_3_ exhibits green emission, enabling decryption. Subsequently, they utilize UCNP@CsMnCl_3_ to make more complex graphs, and use CsMnCl_3_ to fill the blank space, then encryption mode with high encryption level could be obtained. It is concluded that UCNP@CsMnCl_3_ are undoubtedly suitable for high-level anti-counterfeiting and high-capacity information encryption.

UCNPs/CDs (CDs = carbon dots) dual-modal luminescent materials have attracted extensive attention^159,160^. Xu et al. ^160^ prepared UCNPs@CDs@mSiO_2_ nanocomposites that can produce red, green, and blue UCL (980 nm laser irradiation) and blue DSL (365 nm light excitation). The nanocomposites could be further manufactured into different luminescent inks to produce highly safe anti-counterfeiting barcodes. Besides, luminescent composites with DSL and UCL such as Gd_2_O_3_:Yb^3+^/Er^3+^/Eu(DBM)_3_Phen^161^, YVO_4_:Er^3+^,Yb^3+^@YPO_4_:Eu^3+^ ^162^, and Y_2_O_3_:Er^3+^,Yb^3+^@SiO_2_@HPU-19b@Eu^3+^(Tb^3+^)^163^, have been used as dual-modal fluorescent inks and barcode, which are potential nanomaterials for anti-counterfeiting.

### Photocatalysis

Recently, the preparation of photocatalysts with broad-spectrum (UV to NIR range) absorption properties to realize the effective utilization of solar energy in various fields (photocatalytic hydrogen production, elimination of environmental pollutants, antibacterial, etc.) has been a hot topic of research^164–168^. Upconversion luminescent materials could absorb NIR light and convert it into UV/Vis light. Therefore, the photocatalysts can be constructed by combining the upconversion material and semiconductor. The resulting nanocomposites could be excited by NIR light and generate photogenerated holes (h^+^) and electrons (e^−^), which could take advantage of sunlight and enhance the photocatalytic efficiency. A variety of UCNPs/semiconductor nanocomposites have been fabricated, such as UCNPs/TiO_2_, UCNPs/CdS, UCNPs/ZnO, etc. complex systems.

#### UCNPs/TiO_2_

**Fig. 10:**
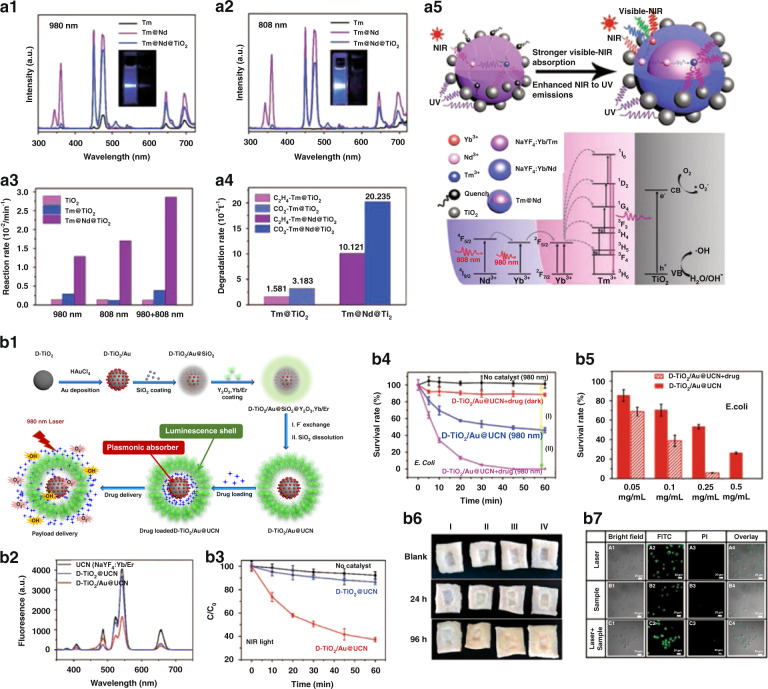
Photocatalysis. UCL spectra of NaYF_4_:Yb^3+^,Tm^3+^ (Tm), Tm@Nd, and Tm@Nd@TiO_2_ under 980 nm excitation (**a1**) and 808 nm excitation (**a2**). The insets of **a1** and **a2** are luminescence photographs of Tm@Nd (left) and NaYF_4_:Yb^3+^,Tm^3+^ (right) with the same concentration dispersed in cyclohexane under 980 and 808 nm excitation, respectively. Insets of **a1** and **a2**: UCL photographs of Tm@Nd (left) and NaYF_4_:Yb^3+^,Tm^3+^ (right) under 980 and 808 nm laser excitation, respectively. **a3** The degradation rates of Rhodamine B by different samples under excitation at 980, 808, and 980 + 808 nm. **a4** Degradation rates of C_2_H_4_ by Tm@TiO_2_ and Tm@Nd@TiO_2_ photocatalysts. **a5** Schematic diagram of enhanced photocatalytic activity and upconversion photocatalytic mechanism^171^. **b1** Schematic diagram of preparation of drug-loaded D-TiO_2_/Au@UCN nanocomposites and NIR light-controlled drug release. **b2** UCL spectra of UCN, D-TiO_2_@UCN, and D-TiO_2_/Au@UCN. **b3** The time-dependent ratios of *C*/*C*_0_ in the presence of D-TiO_2_@UCN and D-TiO_2_/Au@UCN or without photocatalyst for rhodamine 6G degradation under NIR light irradiation. **b4** E. coli viability treated with D-TiO_2_/Au@UCN and AMP-loaded D-TiO_2_/Au@UCN in the dark or under 980 nm irradiation. **b5** Survival rate of *E. coli* under different sample concentrations under NIR light irradiation for 30 min. **b6** Photographs of the bacterial contamination for pig skin in 96 h. Grouping of infected skin (I) drug-loaded D-TiO_2_/Au@UCN + NIR light, (II) drug-loaded D-TiO_2_/Au@UCN in the dark; (III) NIR light irradiation, and (IV) in the dark. **b7** Confocal fluorescence images of HaCaT cells co-stained with V-fluorescein isothiocyanate and propidium iodide after different treatments^172^. **a1–a5** Reprinted with permission from ref. ^171^ Copyright 2019, Elsevier. **b1–b7** Reprinted with permission from ref. ^172^. Copyright 2020, American Chemical Society

TiO_2_ has been widely studied in photocatalytic degradation of organic and inorganic pollutants due to its high catalytic activity, non-toxic and low cost. However, TiO_2_ could only be excited by UV light resulting from its band gap (3.2 eV), which greatly limits its application of photocatalysis. UCNPs/TiO_2_ nanocomposites could avoid the above problem and be served as the photocatalytic materials irradiated by NIR light^169–174^. Huang et al. ^171^ designed NaYF_4_:Yb^3+^,Tm^3+^@NaYF_4_:Yb^3+^,Nd^3+^@TiO_2_ (Tm@Nd@TiO_2_) nanocomposites for NIR photocatalysis. When Tm@Nd was modified with TiO_2_, UV emission of nanocomposites was greatly reduced irradiated at 980 or 808 nm (Fig. 10a1, a2). The Rhodamine B degradation rate constants of nanocomposites under 980, 808, and 980 + 808 nm laser excitation are 4.40, 5.84 and 9.83 times as high as that of Tm@TiO_2_ under 980 nm excitation, respectively (Fig. 10a3). Under 980 + 808 nm laser excitation, the ethylene degradation rate constant of Tm@Nd@TiO_2_ is 6.4 times higher than that of Tm@TiO_2_ (Fig. 10a4). The enhanced photocatalytic activity of nanocomposites could be attributed to strong NIR absorption of Nd^3+^ and intense upconversion emission of UCNPs (Fig. 10a5). This study provides the route for further improving NIR light-mediated photocatalytic activity of TiO_2_-based upconversion photocatalysts. Song and co-workers^172^ prepared D-TiO_2_/Au@UCN nanocomposites and antibiotic drug ampicillin sodium (AMP) was loaded into D-TiO_2_/Au@UCN, which can be used as NIR-activated photocatalytic platform for bacterial inactivation (Fig. 10b1). Under 980 nm excitation, the UCL of D-TiO_2_/Au@UCN is significantly lower than that of pristine UCN, resulting from the efficient Vis light harvesting of D-TiO_2_@Au (Fig. 10b2). D-TiO_2_/Au@UCN can decompose more than 60% of rhodamine 6G (Fig. 10b3), which is much higher than that of the control and D-TiO_2_/UCN groups (degradation rate: 13.3%). Under NIR-light irradiation, almost no viable *E. coli* could be observed after incubation for 60 min, showing stronger bactericidal activity of AMP-loaded D-TiO_2_/Au@UCN (Fig. 10b4, b5). Such NIR light-triggered system exhibits excellent photocatalytic bactericidal performance under deep tissue penetration conditions (Fig. 10b6). The negligible cytotoxicity of AMP-loaded D-TiO_2_/Au@UCN was verified by cytotoxicity assay (Fig. 10b7). It could greatly expand TiO_2_-based photocatalysis in destroying antibiotic-resistant and heat-resistant microorganisms.

#### UCNPs/ZnO

**Fig. 11:**
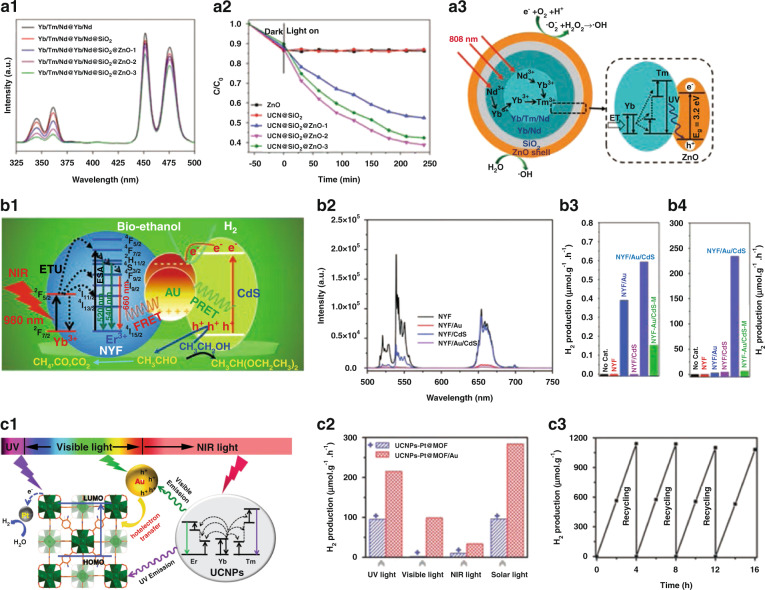
Photocatalysis. **a1** UCL spectra of different samples. **a2** Time-dependent ratios of *C*/*C*_0_ for Rhodamine B with different samples as photocatalysts. **a3** ET process in UCNPs and the proposed photocatalytic mechanism for UCN@SiO_2_@ZnO^176^. **b1** ET processes between NYF, Au, and CdS, and evolution process of bio-ethanol photoreformed H_2_ under NIR irradiation. **b2** UCL spectra of NYF, NYF/Au, NYF/CdS and NYF/Au/CdS. Photocatalytic H_2_ evolution rates of different samples under NIR (**b3**) and simulated sunlight (**b4**)^179^. **c1** Schematic diagram of photocatalytic H_2_ production mechanism of UCNPs-Pt@MOF/Au. **c2** H_2_ production rates of UCNPs-Pt@MOF and UCNPs-Pt@MOF/Au under excitation by UV, Vis, NIR, and solar light. **c3** Recycling test for H_2_ production of UCNPs-Pt@MOF/Au under simulated solar light^164^. **a1–a3** Reprinted with permission from ref. ^176^ Copyright 2020, American Chemical Society. **b1–b4** Reprinted with permission from ref. ^179^ Copyright 2017, The Royal Society of Chemistry. **c1–c8** Reprinted with permission from ref. ^164^ Copyright 2018, John Wiley and Sons

ZnO with a wide bandgap of ~3.37 eV is also widely used in photocatalytic and antibacterial fields. ZnO produces photocatalytic effect under UV light excitation, which restricts its applications in Vis and NIR range. To date, UCNPs@ZnO heterojunction structures have been constructed, which have widened the light response range of ZnO-based photocatalytic materials and improved the photocatalytic activity under NIR light irradiation^175–178^. Li’s group^175^ developed NaYF_4_:Yb/Tm@SiO_2_@ZnO nanocomposites with ideal NIR photocatalytic activity in degradation of organic pollutants and antibacterial activity. Qiao et al. ^176^ prepared NaYF_4_:Yb,Tm,Nd@NaYF_4_:Yb,Nd@SiO_2_@ZnO (UCN@SiO_2_@ZnO), which presented good NIR-induced photocatalytic activity. The UV UCL of UCN@SiO_2_@ZnO could be effectively harvested by ZnO to activate photocatalytic process (Fig. 11a1). UCN@SiO_2_@ZnO shows high degradation rate of Rhodamine B (61.2%) after 808 nm irradiation for 250 min (Fig. 11a2). The upconversion UV emission absorbed by ZnO could effectively generate photogenerated e^−^ and h^+^. With the oxidation–reduction reaction occurs between the substances adsorbed on the surface of ZnO and photogenerated carrier, •OH radicals could be generated to participate in the photocatalytic reaction process as an active substance (Fig. 11a3). This study has important value for developing composite photocatalysts with excellent NIR photoresponsive properties.

#### UCNPs/CdS

CdS with narrow band gap is used to replace TiO_2_ or ZnO and coated on UCNPs, which can make full use of the converted UV and Vis light, improve the utilization of light and enhance the photocatalytic effect^46,179,180^. Li et al. ^46^ developed NaYF_4_:Yb,Tm@C@CdS nanocomposites for NIR-light enhanced photocatalysis. The photocatalytic activity of such nanocomposite was higher than that of CdS and the mixture of NaYF_4_:Yb,Tm and CdS. Liu et al. ^179^ prepared NaYF_4_:Yb/Er@Au@CdS (NYF/Au/CdS) for H_2_ production by photoreforming of bio-ethanol, where Au component promoted the electron–hole separation through FRET and plasmonic resonance energy transfer (PRET) (Fig. 11b1). The UCL of NaYF_4_:Yb/Er significantly reduced after modification with Au or CdS. For NYF/Au/CdS, the upconversion emissions further diminish (Fig. 11b2). NYF/Au/CdS exhibits promoted NIR light-induced photocatalytic bio-ethanol-reforming activity and has the highest H_2_ yield (0.59 μmol g^−1^ h^−1^) compared with NYF, NYF/Au, and NYF/CdS (Fig. 11b3). Furthermore, NYF/Au/CdS exhibits the largest H_2_ evolution rate under simulated sunlight illumination (Fig. 11b4). This work provides new strategy for developing efficient NIR-driven UC photocatalytic systems.

#### Other UCNPs/semiconductor nanocomposite photocatalysts

Recently, Jiang’s group^164^ prepared UCNPs-Pt@MOF/Au with broad-spectrum absorption characteristic. MOF mainly responds to UV light and Au nanoparticles with plasma resonance effect absorb Vis light, while UCNPs convert NIR light into UV and Vis light, which could be captured by adjacent MOF and Au again, to realize the absorption and utilization of composite materials from UV to NIR range (Fig. 11c1). UCNPs–Pt@MOF/Au nanocomposites show considerable H_2_ production rate irradiated by UV, Vis, and even NIR laser (Fig. 11c2). Importantly, UCNPs–Pt@MOF/Au exhibits good recovery performance under simulated sunlight irradiation, and H_2_ production rate do not change significantly during four catalytic cycles of 16 h (Fig. 11c3). This work opens a way to harness NIR light for photocatalysis. g-C_3_N_4_ has become one of the most promising photocatalysts driven by Vis light. Park et al. ^181^ designed UCNPs/g-C_3_N_4_ nanocomposites with photocatalytic activity much better than pure g-C_3_N_4_.

## Conclusions and outlook

In summary, UCNPs-based nanocomposites are versatile candidates to utilize the UCL characteristics and have great potentials in various applications. This review summarizes the main methods for constructing UCNPs-based nanocomposites, and the applications of such nanocomposites in bioimaging, cancer treatments, anti-counterfeiting, and photocatalysis. Notably, although promising advance has been made in the preparation strategies and applications of UCNPs-based nanocomposites, there are still great challenges in the following aspects.

1. The existing synthesis methods for UCNPs-based nanocomposites still have shortcomings and improvements. Self-assembly method often has disadvantages of time-consuming, easy aggregation, weak adsorption, and the structure is easily to be destroyed under the action of some solvents. In-situ growth method often needs to modify or coat polymers or complexes on UCNPs as precursors, and then the precursors act as nucleation and growth centers to induce other nanodots to be grown further. This also motivates us to develop more novel modified materials, which can not only ensure that the luminescence of UCNPs will not be quenched too much, but also pave the way for the further growth of other materials. The epitaxial growth method usually uses toxic, expensive precursors or organic solvents, and the products are hydrophobic, and the synthesis temperature is relatively high. Heteroepitaxial growth method requires more severe conditions, and is impossible to track the reaction process in situ. Thus, it is difficult to expound the reaction mechanism exactly. Other facile methods to synthesize UCNPs-based nanocomposites remain to be optimized and explored.
2. The biological applications of UCNPs-based nanocomposites are still in the preliminary stage of research, and there are many problems to be solved before realizing clinical transformation. It is necessary to further rationally optimize the chemical composition and structure, reasonable particle size, and surface properties of UCNPs-based nanocomposites to construct nanoplatforms with high uniformity and excellent biocompatibility. Most importantly, to quantitatively load functional molecules (such as photosensitive molecules, anticancer drugs, etc.) and achieve the controllable release are of great significance. It cannot be ignored that reducing the biotoxicity, improving metabolic efficiency in vivo, and ensuring the reproducibility of diagnosis and treatment effects are the prerequisites for the future biological applications of UCNPs-based nanocomposites.
3. The development of superior luminescent nanomaterials and high-tech fluorescent anti-counterfeiting technologies is of great significance for the global economy, security, and human health, which has been proven to be a huge challenge. Tunable multicolor, multi-modal luminescent nanocomposites have been achieved by combining UCNPs with other luminescent components, which could greatly improve the anti-counterfeiting level. Although multiple anti-counterfeiting materials could be used simultaneously to impart multiple security features, the implementation process is complex and leads to low efficiency of production. This motivates researchers to develop novel UCNPs-based nanocomposites with multiple anti-counterfeiting features, different identification methods and easy processing to promote their practical applications. To improve the overall photostability of UCNPs-based nanocomposites is an important direction.
4. UCNPs@semiconductor photocatalysts have unique core-shell structure and good photocatalytic activity under sunlight. Although researchers have made great progress in UCNPs-based nanocomposites with certain photocatalytic activity in the UV, Vis and NIR regions by enhancing UCL of UCNPs, introducing semiconductors, and constructing heterostructures, etc., the utilization rate of Vis and NIR light is still unsatisfactory. The following problems need to be considered and solved: (a) The photocatalytic mechanism of nanocomposites needs to be further studied. (b) The photocatalytic efficiency is not satisfactory. The fluorescence intensity of UCNPs needs to be improved. The surface-enhanced fluorescence phenomenon can be generated by introducing nano-precious metals to improve its fluorescence intensity; new fluorescent materials should be developed to absorb much NIR energy and improve the conversion rate.

The design and applications of UCNPs-based nanocomposites are still in the infancy, the research of fundamental theoretical and practical applications will face complex challenges. This requires close collaboration between researchers from different disciplines to overcome the problems. It is believed that UCNPs-based nanocomposites will lead to major changes in life sciences, anti-counterfeiting technology, optoelectronics, energy catalysis, and other fields.
